# Pediatric pharmaceutical interventions in self-medication: a descriptive study in community pharmacies

**DOI:** 10.1186/s12875-023-02180-9

**Published:** 2023-11-06

**Authors:** Sabrina Bedhomme, Hélène Vaillant-Roussel, Philippe Vorilhon, Elodie Lafarge, Bénédicte Pereton, Céline Prunet-Spano, Bruno Pereira, Brigitte Vennat, Chantal Savanovitch

**Affiliations:** 1 Faculty of Pharmacy, University of Clermont Auvergne, Clermont-Ferrand, France; 2 Research Unit ACCePPT, University of Clermont Auvergne, Clermont-Ferrand, France; 3 Department of General Practice, University of Clermont Auvergne, Clermont-Ferrand, France; 4 Present Address: DRCI, CHU Clermont-Ferrand, Clermont-Ferrand, France; 5 ISPB (Institut des Sciences Pharmaceutiques et Biologiques), Claude Bernard Lyon 1 University, Lyon, France

**Keywords:** Community pharmacy, Non prescription Drugs, Self-medication, Pediatrics, Drug-related problems

## Abstract

**Background:**

The practice of self-medication is common but not without risk, especially for vulnerable populations such as the pediatric population. Community pharmacists have an important role of vigilance in dispensing drugs available without a medical prescription, with the possibility of carrying out a Pharmaceutical Intervention (PI) if necessary. The aim of our study was to characterize the Pediatric Pharmaceutical Interventions (PPIs) in self-medication carried out during a spontaneous request for a drug at the community pharmacy.

**Methods:**

We conducted a descriptive study in 139 pharmacies in the Auvergne-Rhône-Alpes region (France). Data were collected from students under the supervision of internship masters in the pharmacy, using the validated GIPAMED (GrId for PhArmaceutical Self-MEDication interventions) notification grid, the first week of each month, from February to May for five years (2017 to 2021). Collected data were entered on a secure university platform.

**Results:**

Of the 3,552 PIs collected, 8,3% (n = 286) were PPIs. Of these PPIs, 35% (n = 100) was generated by requests for optional prescription drugs contraindicated by the pathophysiological condition, 28.3% for drugs requiring a prescription and 20.6% for over the counter drugs not indicated by the symptomatology. Finally, 10% of requests required a referral for a medical consultation. Four Anatomical Therapeutic Chemical (ATC) classes accounted for more than 90% of the requests: respiratory system (39.5%), alimentary tract and metabolism (19.2%), nervous system (11.5%), and musculoskeletal system (10.8%). The most common drugs generating PPIs were: ibuprofen, oxomemazine and combination camphor/essential oils, mainly due to age-related or weight-related contraindication. Paracetamol also generated PPIs frequently, mainly due to problems with drug compliance and more precise infra-therapeutic doses. When these PPIs were dispensed, the pharmacist’s proposed solutions were accepted in 94.8% (n = 271) of the cases.

**Conclusions:**

The community pharmacist has an important role in providing information about medicines and their correct use to patients. Our research shows that this attention benefits vulnerable populations, such as children, even for drugs that are widely used (e.g. paracetamol and non-steroidal anti-inflammatory drugs) or active substances for which there are age-related or weight-related contraindications (e.g. antitussives, camphor combinations).

## Background

According to the World Health Organization, self-medication “consists of a person choosing and using a drug for a condition or symptom that he or she has identified” [1]. The practice of self-medication is less developed in France than in other European countries: 12% of drug sales compared with 23.5% in Europe, including 40% or more in Belgium, Germany and the United Kingdom, but is still common [2]. In 2020, in France, the main sectors of Over The Counter (OTC) drugs sales in value were digestive disorders, pain and respiratory tract [3]. However, self-medication is not without risk. Analgesics, and particularly paracetamol, are among the most widely used drugs in the world [4]. Studies have shown that the use of high doses of paracetamol is the cause of serious liver damage [5]. In 2009, in the United States, the Food and Drug Administration (FDA) was concerned about paracetamol overdoses, the leading cause of acute liver injury, half of which were unintentional overdoses [6] [7]. European countries that allow the sale of certain drugs outside pharmacies, are going backwards. For example, the explosion of paracetamol poisoning in Sweden (40% increase between 2009 and 2013), meanwhile, argued for the pharmacy monopoly; the Swedish Medical Products Agency withdrew most paracetamol presentations from sale in supermarkets on November 2015, citing public health reasons; the dispensing of paracetamol tablets is once again reserved for pharmacies [8]. In France, the National Agency for the Safety of Medicines and Health Products (ANSM) is imposing a warning message about the risk of liver toxicity on paracetamol packaging in 2019 [9]. Since January 2020, medicines containing paracetamol or NSAIDs had no longer OTC status [10].

The risk is even greater in vulnerable populations such as the pediatric population [11]. In children, the pharmacokinetics and pharmacodynamics of drugs differ from those in adults and expose them to an increased risk of developing potentially serious adverse effects (e.g., Reye’s syndrome with aspirin or acute renal failure with ibuprofen in case of dehydration) [12]. They are also particularly vulnerable to drug interactions, misuse or delayed diagnosis or worsening of an underlying pathology (e.g., ibuprofen increases the risk of mucocutaneous complications of varicella [13] [14]. A descriptive observational study of Drug-Related Problems (DRPs) in pediatric population reported to the regional pharmacovigilance center in France in 2016 showed the main prescription or non-prescription drugs involved in “serious” DRPs were paracetamol, asparaginase and ibuprofen [15]. The main ways of ingestion lead to paracetamol intoxication in children are: deliberate overdose, unintentional exposure or administration error. Although children appear to be less sensitive than adults, repeated use of supratherapeutic doses or a massive dose may cause liver damage [16]. In 2023 an Italian study showed that the most common reason for inappropriate oral use of paracetamol or ibuprofen in children was misuse or accidental ingestion. Nausea was the most common adverse effect reported in children taking paracetamol, followed by hypertransaminasemia and vomiting. In children taking ibuprofen, abdominal pain, vomiting, dizziness and nausea (23%) were the most common adverse reactions reported [17].

The community pharmacist plays an important role in “pharmaceutical care”, ensuring the safe and rational use of medicines. Their multiple roles include dispensing of medicines, primary care, patient counselling, health education and prevention [18]. “He has a special duty to advise when he is called upon to dispense a medicine that does not require a medical prescription”, as described in the Public Health Code [19].

Good practice in dispensing medicines in community pharmacies states that during the pharmaceutical analysis of a prescription or a request for a non-prescription drug, “writing a pharmaceutical intervention (PI) is advised when the pharmacist identifies a problem involving the effectiveness or safety of the treatment. This makes it possible to formalize the pharmaceutical analysis in writing and to possibly communicate it to the prescriber” [20]. A PI is defined as “any proposal initiated by the pharmacist to modify the medicinal therapy” or “any activity initiated by the pharmacist that is of benefit to the patient” [21].While prescription PIs are traced to the prescriptions, non-prescription drugs PIs, which are an important part of the community pharmacist’s daily work, are mostly not.

Therefore, there is a paucity of data on self-medication PPIs in the literature. When they do exist, they are hospital data, mostly concerning specific populations (e.g. pediatric intensive care) [22], and prescription-only drugs [23] [24].

The aim of our study was to characterize the Pediatric Pharmaceutical Interventions (PPIs) in self-medication carried out during a spontaneous request for a drug at the community pharmacy. The secondary aim was to show the role that the pharmacist can play in the prevention of non-prescription drug misuses in pediatrics.

## Methods

### Study design

This was a descriptive multicenter study of PPIs in self-medication. Data were collected from 139 pharmacies in the Auvergne Rhône-Alpes region that host a student in the last year of pharmaceutical studies in a professional practice internship. In general population, PIs were collected using a validated reporting grid for self-medication, the GIPAMED (GrId for PhArmaceutical Self-MEDication interventions) reporting grid [25]. All the PIs were issued during the first week of each month, from February to May for five years (2017 to 2021). These data collection periods, included in the students’ internship periods, were chosen in order to keep the data collection systematic during a short period. Data collectors were students under the supervision of internship masters in the pharmacy. A meeting was held beforehand to present the study and train both of them in the use of the reporting grid.

All PIs for self-medication were generated in GIPAMED but only PPIs, i.e. generated following a spontaneous and specific request for a non-prescription drug for a child under 18, were retained in this study. The following were not included in GIPAMED: PIs generated by requests for essential oils alone, dietary supplements, and medical devices, and requests from parents describing their child’s symptoms without spontaneous requests for medication.

### Data collection

Each PPI was captured in the GIPAMED reporting grid, which included the following data: drug involved, patient information (gender, age), reason for the PPI (contraindication, allergy, drug interaction, redundancy, underdose, overdose, non-indication), method of PPI detection (drug history, dialogue), solution proposed by the providers and validated by a pharmacist (dosage adjustment, therapeutic alternative, need for medical consultation, provision of lifestyle and dietary advice), and outcome of the PPI (accepted or rejected by the patient or his representative). The grid also included a free text area where the history of the PPI is described.

### Statistical analysis

The data collectors entered each PPI on a secure university platform of the RENATER national network and shared with the steering committee. The data were exported to Excel® software (Office 2010, Microsoft Corporation, USA) for analysis. Each PI is validated by reference to the details of the PI described in the text box, which allows the PI to be contextualised and checked for consistency with the data entered in the grid. This review is carried out by the steering committee composed of pharmacists, university pharmacists and research professors.

All descriptive statistical analyses were performed with Stata (version 13, StataCorp, College Station, USA) software. The results concerning categorical data were presented with frequencies and 95% confidence intervals.

### Ethics approval and consent to participate

The study was registered with the French Commission on Information Technology (n°2016-13). In accordance with French law (Decree No. 2017 − 884 of May 9, 2017, Art. R. 1121-1.- I.), a positive advisory ethical opinion was obtained from the Committee for the Protection of Persons South Est VI, Clermont-Ferrand (n° 2016/CE17), which waived the need for informed consent. No children were interviewed by the researchers, as the study focused on the professional practice of pharmacists, who reported data collected in the course of their professional practice. This research was conducted in accordance with the Declaration of Helsinki.

## Results

A total of 3,552 PIs were collected from 2017 to 2021, and 8.4% (n = 300) were PPIs. Of the 300 PPIs collected, 14 were excluded from the analysis: medical devices (n = 10), essential oils alone (n = 1) and drugs withdrawn from the market at the time of data analysis (n = 3). Thus, 286 PPIs were included in the statistical analysis. Patient characteristics are shown in Table 1.

**Table 1:** Pediatric Pharmaceutical Interventions in self-medication: Characteristics of the study population (applicant, gender, age) (n = 286)

Characteristic	n	%	95% IC
*Applicant*			
Patient himself	**26**	**9.1%**	[5.8 − 12.4%]
A representative	**260**	**90.9%**	[87.6 − 94.2%]
Child gender*			
Boy	**143**	**50.0%**	[44.2 − 55.8%]
Girl	**142**	**49.7%**	[43.9 − 55.4%]
*Child age*			
≤ 3 years	**105**	**36.7%**	[31.1 − 42.3%]
3–14 years	**153**	**53.5%**	[47.7 − 59.3%]
15–17 years	**28**	**9.8%**	[6.3 − 13.2%]

*No response = 1

In 98.6% of cases (n = 282), PPIs were identified through dialog with an interviewer. In 2.8% of cases (n = 8), the dialogue was accompanied by a consultation about the medication history.

### Main ATC classes concerned by PPIs

The distribution of PPIs by ATC class is shown in Table 2.

Four ATC classes accounted for more than 90% of PPIs:

- respiratory system drugs (38.8%);
- digestive tract and metabolism drugs (19.2%), mainly diarrhoea drugs (47.3%) and antiemetics/antinauseants (23.6%);
- nervous system drugs (11.5%): mainly analgesics (69.7%) or local anesthetics (18.2%);
- musculo-skeletal drugs (10.8%): mainly anti-inflammatory drugs (74.2%), 16.1% were related to topical anti-inflammatory drugs.

**Table 2:** Distribution of pediatric pharmaceutical interventions by Anatomical Therapeutic Chemical classes

Anatomical Therapeutic Chemical classes	n	%	95% IC
Respiratory system	**111**	**38.8**	[33.2 − 44.5%]
Alimentary tract and metabolism	**55**	**19.2**	[14.7 − 23.8%]
Nervous system	**33**	**11.5**	[7.8 − 15.2%]
Musculo-skeletal system	**31**	**10.8**	[7.2 − 14.4%]
Dermatologicals	**29**	**10.1**	[6.6 − 13.6%]
Sensory organs	**13**	**4.5**	[2.1 − 7.0%]
Genito urinary system ans sex hormons	**4**	**1.4**	[0.0 − 2.8%]
Antiinfective for systemic use	**3**	**1.0**	[0.0 − 2.2%]
Antiparasitic products, insecticies ans repellents	**3**	**1.0**	[0.0 − 2.2%]

*No response = 4

### Reasons for PPIs and main active substances concerned by PPIs

#### Reasons for PPIs

35% (n = 100) of PPIs were generated by requests for optional prescription drugs contraindicated by the pathophysiological condition, including allergy, 28.3% by requests for prescription drugs, 20.6% by requests for drugs not indicated for the symptomatology, and 10% of requests required referral for medical consultation.

The distribution of the reasons for PPIs and main active substances by reason is shown in Table 3.

#### Main active substances concerned by PPIs

The most common active substance for PPIs were ibuprofen (7.7%, n = 22), paracetamol (7.3%, n = 21), oxomemazine (6.3%, n = 18), camphor/turpentine/levomenthol/eucalyptus essential oil (5.9%, n = 17), racecadotril (4.9%, n = 14) and metopimazine (4.5%, n = 13).

#### Main reasons by active substances

The main reasons by active substance for PPIs were as follows:

For ibuprofen, the main reasons for PPIs were the physio-pathological contraindications (and specifically age-related contraindications) and non-accordance with legislation (i.e. requests for prescription-only medicines).

For paracetamol, the reasons for PPIs were varied, but one main reason emerged: compliance, as the drug is used in subtherapeutic doses (n = 4).

For camphor/turpentine/levomenthol/eucalyptus essential oil (7.7%, n = 18) and oxomemazine (6.7% n = 17), which are two “ear/nose/throat” medications, the most common reason for PPIs was a physio-pathological contraindication especially an age-related contraindication.

For racecadotril and metopimazine, the most common reason for PPIs was non-accordance with legislation (i.e. prescription drugs).

**Table 3:** Reasons for pediatric pharmaceutical intervention in spontaneous requests in descending order and main active substances involved (n ≥ 4)

PPI’s reasons	Active substances	n	%	95% IC
Contraindication with the physio-pathological state		**100**	**35.0**	[29.4 − 40.5%]
	Camphor/turpentine/ levomenthol/ eucalyptus essential oil	**12**		
	Ibuprofen	**12**		
	Oxomemazine	**6**		
Non accordance with legislation		**81**	**28.3**	[23.1 − 33.5%]
	Racecadotril	**12**		
	Metopimazine	**9**		
	Desloratadine	**8**		
Non indication		**59**	**20.6**	[15.9 − 25.3%]
	Oxomemazine	**10**		
	Paracetamol	**4**		
	Alpha-amylase	**4**		
	Disomectite	**4**		
Need for a medical consultation		**30**	**10.5**	[6.9 − 14.0%]
	Ibuprofen	**5**		
	Paracetamol	**4**		
	Phenazone/lidocaine hydrochloride	**4**		
Compliance problem		**10**	**3.5**	[1.4 − 5.6%]
Overdosage risk		**4**	**1.5**	[0.0 − 2.8%]
Already use without effectiveness		**1**	**0.3**	[0.0 − 1.0%]
Drug interaction		**1**	**0.3**	[0.0 − 1.0%]

### Main active substances and main reasons of PPIs by age group

The main active substances affected by PPIs by age group are shown in Table 4.

**Table 4:** Main active substances affected by Pediatric Pharmaceutical Interventions by age group (n **≥** 4)

Age	Main active substances	n	%	95% IC	Main reasons (n ≥ 4)
≤ 3 years		**105**			
	Paracetamol	10	9.6	[3.9 − 15.1%]	Compliance problem
	Camphor/turpentine/levomenthol/eucalyptus essential oil	8	7.7	[2.5 − 12.7%]	Physio-pathological contradication
	Oxomemazine	7	6.7	[1.9 − 11.4%]	Physio-pathological contradication
	Racecadotril	7	6.7	[1.9 − 11.4%]	Non-accordance with legislation
	Diosmectite	6	5.8	[1.3 − 10.2%]	
	Ibuprofen	5	4.8	[1.3 − 10.2%]	
	Metopimazine	5	4.8	[0.7 − 8.8%]	
3–17 years		**181**			
	Ibuprofen	17	9.3	[5.1 − 13.6%]	Physio-pathological contradication
	Oxomemazine	11	6.0	[2.6 − 9.6%]	Non indication
	Paracetamol	11	6.0	[2.6 − 9.6%]	Compliance problem
	Camphor/turpentine/levomenthol/eucalyptus essential oil	9	4.9	[1.8 − 8.1%]	Physio-pathological contradication
	Metopimazine	8	4.4	[1.4 − 7.4%]	
	Acide ascorbique/paracetamol/ pheniramine maleate	7	3.8	[1.1 − 6.7%]	
	Racecadotril	7	3.8	[1.1 − 6.7%]	

In children under 3 years of age, the most common drugs generating PPIs were as follows:

- Paracetamol: the main reason was a compliance problem (40.0% of paracetamol PPIs in children under 3 years, n = 4), i.e. an infratherapeutic dose;
- Camphor/turpentine/levomenthol/eucalyptus essential oil and oxomemazine: the only reason was a physio-pathological contraindication, i.e. an age-related contraindication;
- Oxomemazine: the main reason was a physio-pathological contraindication, i.e. an age-related contraindication;
- Racecadotril: the main reason was non-accordance with legislation.

Among those older than 3 years, our results have shown:

- Ibuprofen: the main reason was contraindication, most often age-related contraindication (58.8%, n = 10), except in the specific population of 15- to 17-year-olds, with dental infection as contraindication;
- Paracetamol: with, as previously described, the problem of compliance (36.4%, n = 4) but also weight-related contraindications (27.3%, n = 3);
- Oxomemazine: the main reason was non-indication (81.8%, n = 9);
- Camphor/turpentine/levomenthol/eucalyptus essential oil: the only reason was the physio-pathological contraindication, especially age-related contraindications.

### Proposed solutions and acceptance level

When dispensing these PPIs, the pharmacist had the option of proposing one or more solutions to the patient: in 68.2% of cases (n = 195), an alternative treatment was proposed, in 32.2% of cases (n = 92), the pharmacist referred the patient for medical consultation, and in 17.8% of cases (n = 51), lifestyle and dietary advices were given.

The solutions proposed by the pharmacist were accepted by the patient or his representative in 94.8% (n = 271) of cases. In the specific case of PPIs generated by a prescription-only drugs request (n = 81), the pharmacist referred the patient for a medical consultation in 49.3% of cases (n = 40) and added a proposal for a therapeutic alternative and/or lifestyle and dietary advice in 27.5% of cases (n = 11).

## Discussion

The aim of our study was to describe PPIs. Thus, the main reasons for PPIs were as follows in descending order: the request for non-prescription drugs contraindicated with the physiopathological state, the request for drugs requiring a prescription, the request for non-prescription drugs not indicated with regard to the symptomatology and the need for a medical consultation. Paracetamol and ibuprofen were the most represented active substances, all PPIs reasons together. Respiratory system drugs accounted for more than 1/3 of the requests with the main reason for related PPIs: age-related contraindications. Among the only-prescription drugs, racecadotril and metopimazine were the most common.

### Main reasons of PPIs, ATC classes and active substances

Overall, our results are comparable to those found in the literature. A 2012 German study investigated the type and frequency of DRPs in self-medication in daily community pharmacy practice. Four indications accounted for more than 70% of all DRPs: pain, respiratory, gastrointestinal, and skin disorders. In 20% of cases, the product requested was inappropriate [26]. In 2016, a study by Panda et al. aimed to identify and compare the nature of the DRPs associated with self-medication and prescription-guided drug use. In the self-medication group, the prevalence of DRPs was high as compared to the non-self-medication group. In the non-self-medication group, pain and fever were the most common indications. In the self-medication group, the most common type of DRPs were problems with drug dosage and problems with drug selection [4]. In 2019, Tesfamariam et al., asked patients about their self-medication practices in pharmacies in Eritrea. The therapeutic classes identified as most commonly used were analgesics, antipyretics and cough and cold preparations. Of these, 7% had experienced DRPs, with ibuprofen identified as the most common cause of DRPs [27]. In 2020, a Finnish study, that investigated PIs in community pharmacies, the main classes generating PIs were: respiratory system, digestive tract and nervous system drugs [28].

### The case of paracetamol and ibuprofen

In our study, paracetamol and ibuprofen were the two most represented active substances. For self-medication, ibuprofen is available for infants over three months of age and paracetamol from birth. It is interesting to note that the main reason for a PPI concerning paracetamol is the use of infratherapeutic doses in children under 3 years, which certainly limits the risk of hepatic complications but may lead to poor management of pain or fever and thus could lead to the use of other less safe drugs, such as ibuprofen. For children over 3 years of age, on the other hand, the main reason is the contraindication related to weight: the dosage required is too high in relation to the child’s weight. As for ibuprofen, this molecule is not among those that induce the most PPIs in children under 3 years old. However, it is the molecule that induces the most PPIs in children over 3 years old, with the main reason being age-related contradiction.

The population’s knowledge of self-medication is limited: for example, in a 2013 study in France, the maximum recommended daily dose of paracetamol and ibuprofen was known by 58% and 18% of the users respectively [29]. In 2015, a French study on good use and knowledge of paracetamol among self-medicating patients showed that most of patients (84%) could be considered as “good users” but 1 in 10 patients exposed themselves to a supratherapeutic dose, 1 in 5 patients potentially exposed themselves to an unintentional overdose situation, and only 1 in 3 patients knew about the risk of liver toxicity [30]. NSAIDs are commonly used despite the presence of contraindications such as cardiovascular diseases or pregnancy and their use carry a risk of ulceration and gastrointestinal bleeding. However, the risk of DRPs in self-medication seems to be moderate due to the short duration of use [31]. A significant relationship has been shown between parents’ beliefs about self-medication and their tendency to administer medication to their children without medical advice [32].

### The case of oxomemazine and camphor-based combination

Oxomemazine is contraindicated in children younger than 36 months [33]. In our study we observe age-related contraindication as the main reason in children aged 3 years of age or less. The effect of PPIs is significant because phenothiazines are considered to be possible risk factors in the occurrence of sudden infant death syndrome [34]. The same is true for the camphor-based combination for bronchial decongestion, which is strictly contraindicated before 6 years of age due to the presence of terpene derivatives that lower the seizure threshold [35].

### The case of racecadotril and metopimazine

Data from the literature show that parents most often self-medicate their children with previously prescribed drugs, particularly antipyretics and antitussives, which are drugs found in those who have induced PPIs [32] [36]. In France, racecadotril is only available on prescription for children, and the only non-prescription form of metopimazine is contraindicated before the age of 6 years [37] [38].

### Solutions suggested by community pharmacists

In our study, solutions proposed by the pharmacist were accepted in 94.8% of cases. Even when the pharmacist refers to a medical consultation, he or she also proposes a therapeutic alternative or lifestyle and dietary advice in one third of the cases.

In 2016, Westernlund et al. described the most common ways of answer to a problem in general population by Swedish pharmacists i.e. consumer drug counselling (61.1%), drug switching (43.9%), and referral to a physician (27.5%) [39]. In 2017, Abraham et al. showed, in a study conducted in Pennsylvania, that in more than 2/3 of the cases, the pharmacist suggested a therapeutic alternative that was almost always accepted by the interlocutor, and in 1/3 of the cases, he referred the patient for a medical consultation [40].

### The role of the community pharmacist

Pharmacist intervention in request for non-prescription drug requests is essential, as it has been shown that children who are self-medicate in the first years of life are more likely to continue this practice in adolescence and adulthood, including self-medication with analgesics such as paracetamol [41] [42].

Among the drugs most commonly used by adolescents, who are often autonomous in their self-medication, are NSAIDs and antitussives [43] [44]. These drugs are easily available for the treatment of benign pathologies; however, they expose children to several risks, such as incorrect self-diagnosis, noncompliance with contraindications, overdose or the occurrence of adverse effects [45]. Information on the correct use of the medication, provided to the parents or ideally to the child himself, will ensure current and future use of medication.

The pharmacist must also ensure that the parents have understood the use of the administration devices for the pediatric forms, which are mostly liquids: oral syringes, dosing cups, cylindrical spoons, which are different for each drug and may cause confusion and lead to in use of the medicine. If necessary, he refers the patient to another part of the health care system.

In most European countries, pharmacies do not have a monopoly on the distribution and dispensing of medicines, the non-prescription drugs can also be sold elsewhere, for example in supermarkets, grocery stores, drugstores… In some countries, such as Italy or Portugal, OTC drugs can be sold in supermarkets under the supervision of a pharmacist [46]. The particularity of the French model is that the monopoly of dispensing medicines by pharmacists in community pharmacies is based on a monopoly of competence: the public authorities reserve the sale of medicines to pharmacists and in return, the pharmaceutical activity is subject to strict control [47]. This monopoly aims to control and secure the use of medicines, particularly in self-medication. The pharmacist is the guarantor of the correct use of the medication especially for vulnerable populations such as children. The implementation and collection of PPIs are indicators of this expertise. The lack of legibility of the pharmaceutical act in self-medication could lead to a reconsideration of the pharmaceutical monopoly [48].

### Strengths and limitations

The originality of this study lies in its focus on the pediatric population.

Although the Auvergne Rhône-Alpes region is geographically a large region with both urban and rural areas, the PPIs were collected in 139 French pharmacies located in only one region and are therefore not representative of the nation. However, similar results are found in studies from other countries, which limits that bias.

Data collection took place during the first week of each month, from February to May, which may lead to an overestimation of the number of PIs concerning respiratory drugs due to a seasonal bias. In addition, the collection of PIs in 2020 and 2021 was affected by the COVID-19 pandemic, which may have modified both the quantity and the quality of requests for self-medication at pharmacies. The decree of March 2020 imposed a dispensation of paracetamol without prescription limited to two boxes for patients reporting symptoms of fever or pain and one box in other cases [49] and the High Council of Public Health advised against the use of NSAIDs to treat the symptoms of COVID-19: these 2 elements have probably led to a lower demand for these active substances during this period.

## Conclusions

Although the practice of self-medication is common in the general population and in children, this does not mean that it is safe. Patients often misunderstand contraindications and dosages.

The pharmacist has an important role to play in providing patients with information about medicines and how to use them correctly. Special attention must be paid to vulnerable populations, such as children, even with prescribed and widely used drugs such as paracetamol and NSAIDs, or with active substances that have age- and weight-related contraindications.

In addition, this study highlights the role of community pharmacists as an essential link in the safety of drug dispensation, particularly in self-medication and the high level of patient acceptance of PPIs enhance the role of the pharmacist.

A national study using the same PPIs survey tool would be interesting to confirm our results. Further studies should be conducted to evaluate the level of risk averted by the community pharmacist’s intervention.

## Data Availability

The datasets used and/or analysed during the current study are available from the corresponding author on reasonable request.

## Abbreviations

PI: Pharmaceutical Intervention
PPIs: Pediatric Pharmaceutical Interventions
GIPAMED: GrId for PhArmaceutical self-MEDication interventions
ATC: Anatomical Therapeutic Chemical
OTC: Over The Counter
NSAIDs: Non-Steroidal Anti-Inflammatory Drugs
DRPs: Drug Related Problems
